# A Structural and Mutagenic Blueprint for Molecular Recognition of Strychnine and *d*-Tubocurarine by Different Cys-Loop Receptors

**DOI:** 10.1371/journal.pbio.1001034

**Published:** 2011-03-29

**Authors:** Marijke Brams, Anshul Pandya, Dmitry Kuzmin, René van Elk, Liz Krijnen, Jerrel L. Yakel, Victor Tsetlin, August B. Smit, Chris Ulens

**Affiliations:** 1 Laboratory of Structural Neurobiology, KULeuven, Leuven, Belgium; 2 Laboratory of Neurobiology, National Institute of Environmental Health Sciences, National Institutes of Health, Department of Health and Human Services, Research Triangle Park, North Carolina, United States of America; 3 Department of Molecular Basis of Neurosignaling, Shemyakin-Ovchinnikov Institute of Bioorganic Chemistry, Russian Academy of Sciences, Moscow, Russia; 4 Department of Molecular & Cellular Neurobiology, Center for Neurogenomics and Cognitive Research, Neuroscience Campus Amsterdam, VU University, Amsterdam, The Netherlands; University of Zurich, Switzerland

## Abstract

Cys-loop receptors (CLR) are pentameric ligand-gated ion channels that mediate fast excitatory or inhibitory transmission in the nervous system. Strychnine and *d*-tubocurarine (*d-TC*) are neurotoxins that have been highly instrumental in decades of research on glycine receptors (GlyR) and nicotinic acetylcholine receptors (nAChR), respectively. In this study we addressed the question how the molecular recognition of strychnine and *d-TC* occurs with high affinity and yet low specificity towards diverse CLR family members. X-ray crystal structures of the complexes with AChBP, a well-described structural homolog of the extracellular domain of the nAChRs, revealed that strychnine and *d-TC* adopt multiple occupancies and different ligand orientations, stabilizing the homopentameric protein in an asymmetric state. This introduces a new level of structural diversity in CLRs. Unlike protein and peptide neurotoxins, strychnine and *d-TC* form a limited number of contacts in the binding pocket of AChBP, offering an explanation for their low selectivity. Based on the ligand interactions observed in strychnine- and *d-TC*-AChBP complexes we performed alanine-scanning mutagenesis in the binding pocket of the human α1 GlyR and α7 nAChR and showed the functional relevance of these residues in conferring high potency of strychnine and *d-TC*, respectively. Our results demonstrate that a limited number of ligand interactions in the binding pocket together with an energetic stabilization of the extracellular domain are key to the poor selective recognition of strychnine and *d-TC* by CLRs as diverse as the GlyR, nAChR, and 5-HT_3_R.

## Author Summary

Ligand-gated ion channels play an important role in fast electrochemical signaling in the brain. Cys-loop receptors are a class of pentameric ligand-gated ion channels that are activated by specific neurotransmitters, including acetylcholine (ACh), serotonin (5-HT), glycine (Gly), and γ-aminobutyric acid (GABA). Each type of cys-loop receptor contains an extracellular domain that specifically recognizes only one of these four neurotransmitters and opens an ion-conducting channel pore upon ligand binding. In this study, we investigated the poor specificity with which two potent neurotoxic inhibitors, namely strychnine and *d*-tubocurarine, are recognized by different cys-loop receptors. Using X-ray crystallography we solved 3-dimensional structures of strychnine or *d*-tubocurarine in complex with ACh binding protein (AChBP), a well-recognized structural homolog of the nicotinic ACh receptor. Based on ligand-receptor interactions observed in AChBP structures we designed mutant GlyR and α7 nAChR to identify hot spots in the binding pocket of these receptors that define potent inhibition by strychnine and *d*-tubocurarine, respectively. Combined, our results offer detailed understanding of the molecular recognition of antagonists that have high affinity but poor specificity for different cys-loop receptors.

## Introduction

**Figure 1:**
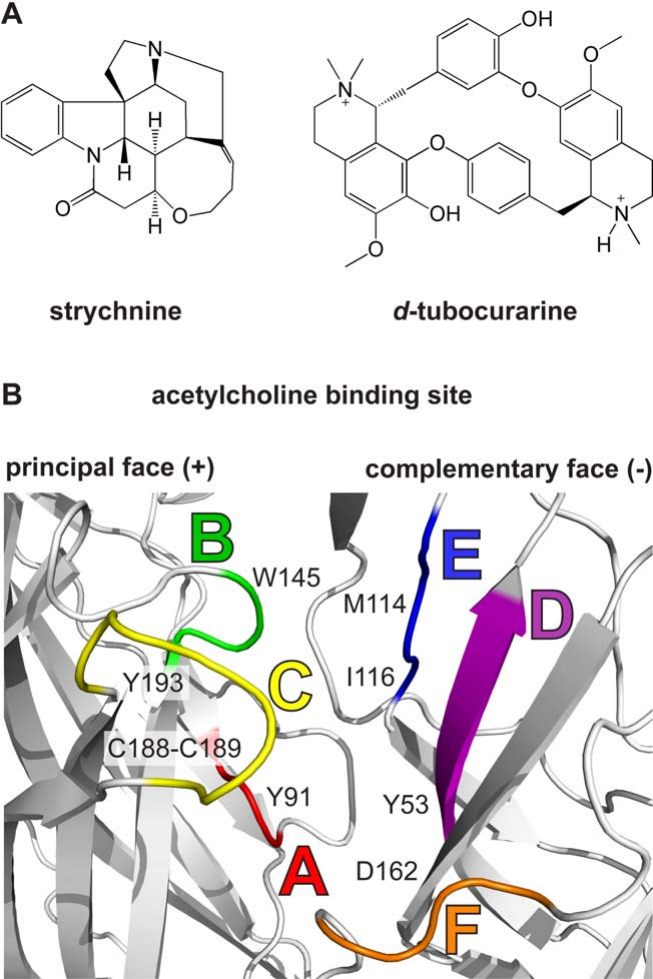
Introduction. (A) Structure formulas of strychnine and *d*-tubocurarine (*d-TC*). (B) Cartoon representation of the acetylcholine binding site in AChBP, which consists of the principal (+) face and complementary (−) face. Different colors are used to highlight loops that form the binding site: loop A (red), B (green), C (yellow), D (magenta), E (blue), and F (orange). Individual residues contributing to loops A–F are indicated according to their numbering in *Aplysia* AChBP.

Strychnine and *d-TC* (Figure 1A) are alkaloids from poisonous plants. Strychnine exerts its lethal effects by antagonizing inhibitory glycine receptors (GlyR) in the central nervous system. Intoxication with strychnine causes muscle spasms, convulsions and eventually leads to death by respiratory paralysis. Clinical use of strychnine is restricted, but it is still applied as a rodenticide. Unlike strychnine, curare is not a homogenous substance but a cocktail of compounds derived from different plant families. One of the best-described active compounds is *d*-tubocurarine (*d-TC*), a quaternary head-to-tail tetrahydroisoquinoline that potently antagonizes the action of acetylcholine on muscle-type [1],[2] and neuronal [3],[4] nAChRs. Intoxication leads to complete paralysis of all skeletal muscles and death by respiratory paralysis. In the Western world *d-TC* analogs have been used in anesthesia as a muscle relaxant during surgery.

In addition to their clinical use *d-TC* and strychnine have been essential molecular tools for the pharmacological characterization of different cys-loop receptors (CLR). *d-TC* and strychnine act as competitive antagonists with very high affinity for nAChRs and GlyRs, respectively. However, their actions extend to other members of the CLR family. For example, *d-TC* antagonizes the action of serotonin on 5-HT_3_ receptors [5],[6]. Strychnine mainly blocks the inhibitory GlyR but also antagonizes certain GABA_A_ receptors [7] and nAChRs [8],[9]. This mode of action strikingly differs from that of protein and peptide neurotoxins such as α-bungarotoxin and α-conotoxins, which in general bind with high affinity and specificity to distinct subtypes of nAChRs, and not to other CLRs.

Our understanding of the molecular action of *d-TC* and strychnine derives from decades of research including ligand competition assays, receptor labeling, electrophysiological studies, and site-directed mutagenesis [1],[2],[5],[10]–[19]. Mutational analysis of the homomeric α1 GlyR revealed several residues in the extracellular ligand-binding domain important for agonist and antagonist binding (reviewed in [20],[21]). Additional evidence for amino acids involved in strychnine binding comes from the identification of a single amino acid substitution in the neonatal-specific α2 GlyR that renders newborn rats insensitive to strychnine poisoning [22]. Recently, Grudzinska et al. described the contribution of several key residues to strychnine binding in the β-subunit of heterooligomeric α1β GlyR [23]. Mutational analysis of conserved aromatic residues of nAChRs demonstrated their importance for binding of curariform antagonists [1],[10]. Recently, Gao et al. [24] characterized an extensive set of mutants in acetylcholine binding protein (AChBP), a structural and functional homolog of the extracellular domain of the nAChR (Figure 1B) [25]. Mutagenesis experiments in AChBP [24] and muscle-type nAChR [26] were based on the ligand-receptor contacts observed in docking simulations of *d-TC*- and metocurine-complexes with AChBP.

In this study we addressed a question that is fundamental to CLR function and that is how the binding cavities of CLRs as diverse as the nAChR, GlyR, and 5-HT_3_R recognize inhibitors such as strychnine and *d-TC* with high affinity but low specificity. In particular, we investigated the molecular determinants of ligand recognition of these inhibitors. For this, we co-crystallized AChBP with *d-TC* and strychnine. These structures enabled identification of the ligand-binding modes and contacts formed in the receptor pocket and, complemented with computational simulations, revealed the dynamic effects of antagonist binding. Mutagenesis and electrophysiological recordings of human GlyRs and nAChRs were then used to test the functional relevance and predictive value of these models. Together, our study provides a blueprint for the molecular recognition of poorly selective alkaloid antagonists at different CLRs.

## Results

### X-Ray Crystal Structures of *Aplysia* AChBP in Complexes with d-TC and Strychnine

To investigate the validity of AChBP as a model to understand binding of strychnine and *d-TC* to CLRs we determined the affinity of these ligands for *Aplysia californica* AChBP (Ac-AChBP) [27], a preferred homolog for structural studies. From competitive binding assays with ^3^H-epibatidine and ^3^H-methyllycaconitine we calculated K_i_-values for strychnine and *d-TC* (Table 1). The affinity of strychnine for Ac-AChBP (K_i_ = 38.0±3.3 nM) is more than 100-fold higher than for α7 nAChR (K_i_ = 4,854±133 nM) and is actually close to the high affinity of strychnine reported for the α1 GlyR (K_i_ = 16±2 nM). This suggests that AChBP is an appropriate model to predict binding of strychnine to the nAChR as well as to the GlyR. Similarly, we found that the affinity of *d-TC* for Ac-AChBP (K_i_ = 509.2±38.0 nM) is in the same range as the reported values for binding of *d-TC* at muscle nAChR [1] and the mouse 5-HT_3_R [28]. Together, the high affinity binding of strychnine and *d-TC* makes AChBP suitable for structural studies of ligand-binding modes of these ligands by X-ray crystallography.

**Table 1:** Binding properties of strychnine and *d-TC* on different CLRs.

	K_i_ Strychnine (nM)	K_i_ *d*-Tubocurarine (nM)
Ac-AChBP	38.0±3.3	509.2±38.0
Ls-AChBP	223.5±26.3	170.7±18.3
*human* α7 nAChR	4,854±133	2,975±378
*human* α1 GlyR	16±2^a^	ND
*mouse* 5-HT_3_R	ND	138±22^b^

Binding constants were determined using competitive binding assays with ^3^H-epibatidine or ^3^H-methyllicaconitine (see Text S1). ND, not determined.

a,b Binding constants for strychnine on human α1 GlyR (^a^) and d-TC on mouse 5-HT_3_R (^b^) were taken from previous studies [28],[40].

**Figure 2:**
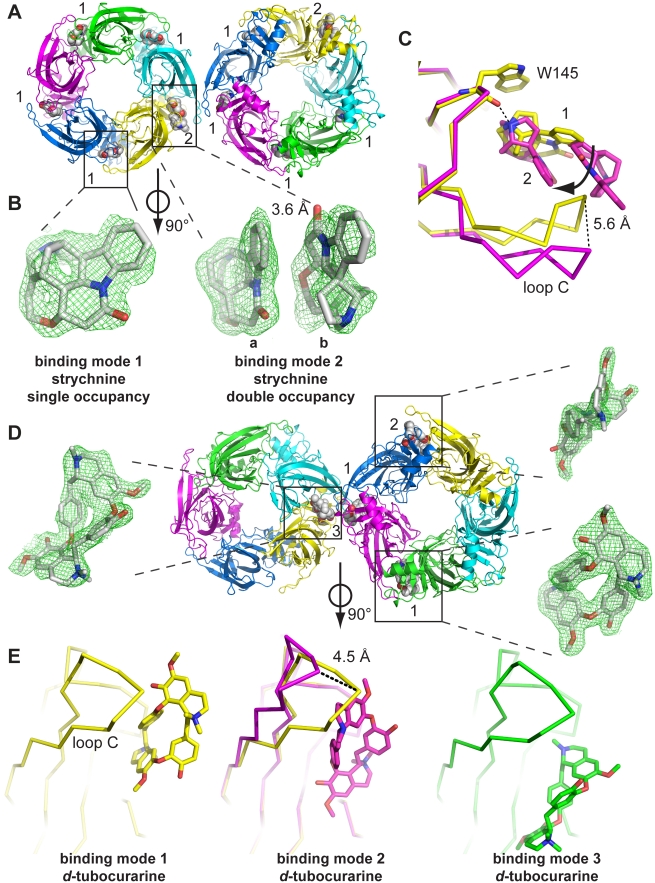
X-ray crystal structures of AChBP complexes with strychnine and *d*-tubocurarine. (A) Crystal structure of *Aplysia* AChBP in complex with strychnine as seen along the 5-fold symmetry axis. The asymmetric unit contains 1 pentamer, which interacts with a neighboring pentamer in the crystal packing through an interaction that involves 2 C-loops. At one of these C-loops the ligand binding pocket is occupied by 2 strychnine molecules (indicated with ‘2’). All 4 other binding pockets contain a single strychnine molecule (indicated with ‘1’). (B) Electron density for strychnine molecules. 2F_o_−F_c_ density for single occupancy was contoured at 1.5 sigma, and for double occupancy the sigma level was 0.8. Ligands are shown perpendicular to the 5-fold symmetry axis. (C) Superposition of the 2 binding modes that occur in the AChBP-strychnine complex. The yellow model shows a detailed view of a principal subunit with a single strychnine molecule bound, and the magenta model shows occupancy by two strychnine molecules. Double occupancy results in an outward movement of loop C by 5.6 Å and a rotation of one strychnine molecule around the N-atom involved in the hydrogen bond with the W145 carbonyl. (D) Crystal structure of *Aplysia* AChBP in complex with *d-TC* as seen along the 5-fold symmetry axis. The asymmetric unit contains 2 pentamers, which also interact through an interface formed by 2 neighboring C-loops. The 10 ligand binding pockets are characterized by the occupancy of *d-TC* molecules in 3 different binding orientations. The predominant binding mode is binding mode 1, which is present in most binding pockets, but with varying degree of ligand occupancy. Only sites with full occupancy of binding mode 1 are indicated with ‘1.’ Ligands in binding modes 2 and 3 have full occupancy and occur only once. Electron density for *d-TC* molecules is shown as 2F_o_−F_c_ density contoured at a sigma level of 1. (E) Comparison of different ligand orientations for *d-TC* relative to the principal subunits. Binding mode 1 is shown in yellow, mode 2 in magenta, and mode 3 in green. Occupancy of *d-TC* in binding mode 2 results in a relative outward displacement of loop C by 4.5 Å compared to binding mode 1.

Crystallization of *Aplysia* AChBP with strychnine or *d-TC* gave co-crystals that diffracted at 1.9 Å and 2.0 Å resolution, respectively, and provided a highly detailed view of their binding modes. Crystallographic data are reported in Table S1. The crystal structure of *Aplysia* AChBP in complex with strychnine contains 1 pentamer in the asymmetric unit (Figure 2A). Similar to other AChBP co-crystal structures (e.g. pdb code 2c9t) the symmetry packing in this crystal form is characterized by an interaction of neighboring pentamers through an interface formed by two C loops (Figure 2A). Inspection of simple difference electron density unambiguously revealed the ligand orientation in all five binding sites of the pentamer (Figure 2B). Remarkably, one of the two C loops involved in forming a crystal contact is in a more extended conformation and reveals electron density for a second strychnine molecule in the same binding site. The second strychnine molecule has a B-factor = 60.67 Å^2^ compared to an average B-factor = 26.24 Å^2^ for all five other strychnine molecules, indicating that the additional strychnine molecule has a more disordered binding mode. The first strychnine molecule, which contacts the (+) face, is pivoted by 48° around the N-atom relative to the strychnine molecule in all other four binding sites (Figure 2C, single occupancy in yellow and double occupancy in magenta). The second strychnine molecule, which contacts the (−) face, is stacked onto the first molecule in a mirrored upside-down orientation and both molecules are separated by a distance of 3.6 Å (Figure 2B). In the binding site with double occupancy, the tip of loop C is displaced outward by a distance of 5.6 Å relative to the binding sites with single occupancy (Figure 2C).

Together, these results show that strychnine molecules, which have a rigid structure, can adopt very distinct but fixed binding orientations in each of the five binding pockets. For the first time we observe the presence of two ligands in the binding pocket of AChBP. Because the C-loop interacting with 2 strychnine molecules also interfaces with a C-loop from a neighboring pentamer the double strychnine occupancy might be the result of a crystal contact. However, interactions between neighboring pentamers comparable to those observed for AChBP may also occur under physiological conditions for intact GlyR and nAChR since these receptors are densely clustered at the neuronal synapse [29],[30].

We also determined the co-crystal structure of *Aplysia* AChBP in complex with *d-TC* in order to verify whether multiple occupancies and distinct ligand orientations are a common property among these alkaloid antagonists. The structure of this complex was solved from diffraction data to 2.0 Å. The asymmetric unit contains two pentamers, which interact through loop C and form an interface that resembles the one seen in the strychnine complex (Figure 2D). Inspection of simple difference electron density revealed occupancy of most of the binding sites by *d-TC*. Remarkably, at least three different binding orientations of *d-TC* can be distinguished (indicated with binding mode 1–3, Figure 2D). Binding mode 1 (yellow, Figure 2E) occurs at most binding sites, but with ligand occupancies that vary between 30% and 60%. d-*TC* molecules were not built in binding sites if the electron density indicated partial occupancy. The binding orientation for these ligands likely corresponds to binding mode 1 but were left unlabeled in Figure 2D. In binding mode 1, the tertiary amine group of *d-TC* forms a hydrogen bond with the carbonyl oxygen of W145 and forms cation-π interactions with conserved aromatic residues of the binding pocket.

A second ligand orientation (mode 2, magenta, Figure 2E) occurs only once in the pentamer and is characterized by a polar interaction between the quaternary amine group of *d-TC* and the carbonyl oxygen of W145. In addition to the upside-down orientation relative to binding mode 1, this ligand is also rotated by 74° toward the (+) face. A superposition of the (+) face in binding mode 1 (protein shown in yellow, Figure 2E) and the (+) face in binding mode 2 (protein and ligand shown in magenta, Figure 2E) shows that the different ligand orientation in mode 2 results in an outward displacement of the tip of loop C by a distance of 4.5 Å relative to mode 1. Finally, difference electron density at one of the binding sites involved in a crystal contact between neighboring C loops indicates the occupancy by a single ligand likely adopting multiple binding orientations. This ligand could be acceptably built into the clearest part of the density and is represented as binding mode 3 (shown in green, Figure 2E). This ligand is rotated by 140° around the tertiary isoquinoline moiety relative to *d-TC* in binding mode 1.

Together, these data demonstrate that rigid molecules like *d-TC* and strychnine can adopt different binding orientations in each of the five equivalent binding sites of the receptor. Loop C, which forms part of the binding site, adopts a more contracted or extended conformational state depending on the binding orientation of the ligand, also when not involved in a crystal contact. Remarkably, this stabilizes AChBP in a structurally asymmetric state even though this pentameric protein is composed of identical subunits. These data imply that potentially a level of functional diversity of CLRs may arise, which depends on receptor occupancy, and that would add to diversity arising from homomeric and heteromeric assemblies of the α- and non-α subunits bearing intrinsic pharmacological differences.

**Figure 3:**
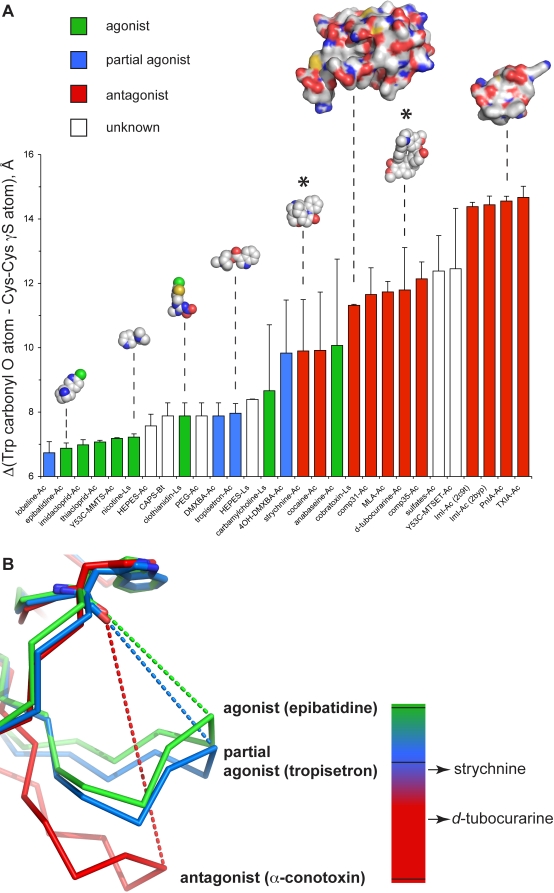
Comparative analysis of C-loop conformation. (A) For each AChBP crystal structure determined to date we quantified the closure of loop C as the distance between the carbonyl oxygen of the conserved Trp residue of loop A and the sulfur atom of the first cysteine involved in the vicinal disulfide bridge formation (W145 and C188 in Ac-AChBP, W143 and C187 in Ls-AChBP, and W142 and C186 in Bt-AChBP). Bars are colored according to the mode of action of each ligand on nAChRs (agonist, green; partial agonist, blue; antagonist, red; unknown, white). Space fill models are shown for selected ligands. Surface representations were used for α-cobratoxin and α-conotoxin PnIA (A10L, D14K). C-loop conformation for strychnine and *d-TC* complexes are indicated with *. (B) Superposition of crystal structures of a prototype agonist (epibatidine), partial agonist (tropisetron), and antagonist (α-conotoxin). The dashed lines indicate the distance measure, which is plotted in panel A. The scale bar on the right gives a visually intuitive interpretation of the mode of action for a compound based on C-loop closure. The C-loop contraction for the 2 co-crystal structures reported in this study is indicated with arrows.

The conformational state of loop C and its contraction around the ligand was quantified by measuring the distance between the carbonyl oxygen atom of W145 and the γ-sulfur atom of C188 in each subunit of the pentamer. In the strychnine complex this average distance is 9.90±1.59 Å, compared to 11.80±1.32 Å in the complex with *d-TC* (indicated with an asterisk in Figure 3A). For epibatidine, an agonist for the nAChRs, this distance is 6.88±0.16 Å and for α-conotoxin ImI, a subtype-specific antagonist for nAChRs, this distance is 14.38±0.13 Å (Figure 3A and 3B). A comparative analysis of C-loop conformations for all agonists, partial agonists, and antagonists currently co-crystallized with AChBP reveals several features. First, for most ligands a correlation exists between the extent of C-loop closure observed in AChBP structures and the ligand mode of action at nAChRs. Ligands that act as antagonists (shown in red bars) typically displace loop C outward by a distance of 10–15 Å relative to the conserved Trp of loop A, whereas agonists (shown in green bars) cause a contraction of loop C around the ligand and reduce this relative distance to <8 Å. Second, partial agonists cause an intermediate contraction of 8–10 Å and typically induce larger variations in the extent of C-loop contraction in different subunits of the pentamer when compared to full agonists and antagonists. This variation can at least in part be explained by the occurrence of different ligand orientations for partial agonists [31]. Third, the position of the C-loop is not a strict predictor for the ligand mode of action at nAChRs because the C-loop closure for some partial agonists overlaps the diffuse boundaries that define agonists versus antagonists (e.g. DMXBA and 4OH-DMXBA). Additionally, lobeline, which acts as a partial agonist at nAChRs [32], causes an exceptionally strong C-loop closure. Together, this comparative analysis rationalizes results obtained from more than 30 co-crystal structures of AChBPs determined to date and shows in general a good correlation between C-loop contraction and predicted ligand action at nAChRs. Finally, multiple ligand orientations are not an exclusive property of partial agonists as suggested by Hibbs et al. (2009) [31] because antagonists such as strychnine and *d-TC* also show multiple conformations.

### Detailed Analysis of the Ligand-AChBP Contacts and Functional Characterization of Contact Mutants in Human CLRs

**Figure 4:**
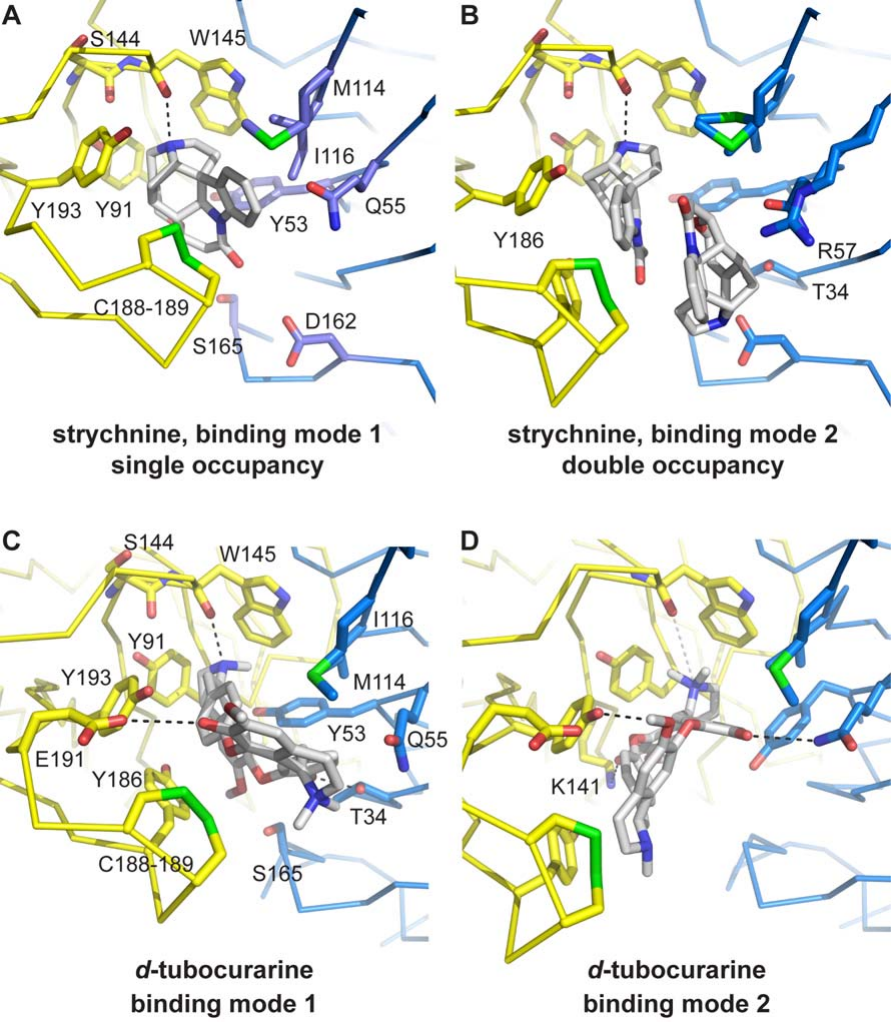
Structural recognition of strychnine and *d*-tubocurarine in AChBP. (A) Detailed view of the ligand binding pocket for single occupancy by strychnine. Principal face (yellow) and complementary face (blue) are shown in ribbon representation. Amino acids involved in ligand-receptor contacts are shown in sticks. (B) Same as in (A) for double occupancy by strychnine. The same amino acids as in (A) are involved in contacts, except for 3 additional contacts formed by Y186, R57, and T34. (C) Detailed view of the amino acids involved in ligand-receptor interactions with *d-TC* in binding mode 1. Principal subunit is shown in yellow ribbon and complementary subunit in blue ribbon representation. (D) Detailed view of amino acids forming ligand-receptor contacts for *d-TC* in binding mode 2. The same residues as in (C) are involved except an additional contact with K141 is formed. Black dashed lines indicate hydrogen bonds. The white dashed line in panel D indicates a polar interaction between the quaternary amine group of *d-TC* and the carbonyl oxygen of W145.

Next, we examined the molecular contacts between ligand and receptor in more detail, and these results were compared for strychnine and *d-TC*. First, strychnine forms contacts with the following residues on the (+) face: Y91, S144, W145, C188, C189, Y193 and (−) face: Y53, Q55, M114, I116, D162, S165 (Figure 4A, Table S2). Three additional contacts are formed in the binding site with double ligand occupancy, namely Y186 on the (+) face and T34, R57 on the (−) face (Figure 4B). In comparison, *d-TC* forms contacts that are remarkably similar. In binding mode 1 (Figure 4C, Table S2), *d-TC* interacts with Y91, S144, W145, C188, C189, Y186, and Y193 on the (+) face. An additional hydrogen bond is formed with E191, an interaction not seen in the strychnine-complex. On the (−) face *d-TC* forms contacts with T34, Y53, Q55, M114, I116, and S165 (Figure 4C). In binding mode 2, *d-TC* forms an additional hydrogen bond with K141 on the (+) face. Due to the different ligand orientations, two hydrogen bonds are formed with Y193 and Q55 (Figure 4D). These findings demonstrate that strychnine and *d-TC*, which differ in chemical and three-dimensional structure, have a large overlap in molecular contacts in the AChBP binding pocket. Also, the set of interactions formed by strychnine and *d-TC* has a much wider range compared to the agonist nicotine (Table S2), which interacts with Y89, W143, T144, C187, and C188 on the (+) face and W53, L112, and M114 on the (−) face (residues and numbering correspond to the *Lymnaea* AChBP complex with nicotine, pdb code 1uw6).

Comparison with residue contacts formed by α-conotoxin ImI, which has high selectivity for distinct nAChR-subtypes (pdb code 2c9t [33]), demonstrates that this peptide antagonist forms residue contacts that overlap with interactions seen for strychnine and *d-TC* but extend to a much wider range of residues (Table S2). α-Conotoxin ImI forms contacts with the (+) face: Y91, S144, W145, V146, Y147, Y186, C188, C189, E191, Y193, and I194 (exclusive contacts are underlined). On the (−) face contacts are formed with Y53, Q55, R57, D75, R77, V106, T108, M114, I116, D162, and S164. Thus, the high subtype-selectivity of α-conotoxin ImI likely arises from a broad range of contacts that are only found in specific nAChR subtypes [33]. In contrast, the poorly selective recognition of strychnine and *d-TC* may arise from similar interactions in binding pockets of different CLRs. For example, residues of loop A and loop C show good sequence conservation among most members of the CLR family. Specifically for loop E, residues homologous to M114 and/or I116 are well conserved between the α7 nAChR (Q117) and 5-HT_3_R (Q123), which are both inhibited by *d-TC*. Good conservation also exists between the α1 GlyR (L127/S129) and α1 GABA_A_-R (L128/T130), which are both inhibited by strychnine.

Are the observed ligand orientations and molecular contacts in AChBP representative for our understanding of antagonist recognition in human CLRs? To address this question, we performed alanine-scanning mutagenesis of the homologous residues in the human GlyR or nAChR using structure-based sequence alignments with AChBP (see Figure S1). The two-electrode voltage-clamp technique was used to measure ligand potency on wild type and mutant GlyR or nAChR expressed in *Xenopus* oocytes. For strychnine, we characterized homologous contact mutants in the human α1 GlyR because it is the primary target that mediates the physiological effects of strychnine. Results of this comprehensive mutagenesis study are summarized in Table 2. We found that strychnine displays a decrease in potency for most α1 GlyR mutants by 1–2 orders of magnitude. F159A and F214A were not functional. Critical contact residues are located in loop B (S158, homologous to S144 in AChBP) and loop D (F63 and R65, homologous to Y53 and Q55 in AChBP, respectively). Mutation of residue S158 in loop B of the GlyR (this study) causes a 300-fold decrease in strychnine-potency, whereas mutation F63A and R65A in loop D [23] cause a 250- and 3-fold decrease in strychnine-potency, respectively. Additionally, mutation of contacts in loop A (A101, homologous to Y91-AChBP) and loop E (L127 and S129, homologous to M114- and I116-AChBP) cause a ∼10-fold drop in potency. Mutation of Q177 in loop F (homologous to D162-AChBP) causes an apparent increase in strychnine-potency. To investigate the relevance of two strychnine molecules occupying a single binding pocket we investigated the effect of mutations at positions homologous to residues involved in unique contacts in the double strychnine occupancy mode, namely T34, R57, and Y186 in AChBP. The IC_50_-values for the homologous mutants in the α1 GlyR, F44A, and Q67A and F207A, are 3-, 15-, and 119,444-fold higher than wild type receptor, respectively. One of these mutants, F207A, was previously characterized in the work from Grundzinska et al. [23]. Such a profound effect of F207A could be expected if the interactions occur as observed for the first strychnine molecule under double occupancy binding mode. The profound effects of these mutations on strychnine inhibition, in particular F207A, suggest that the double occupancy binding mode of strychnine as observed in the crystal structure is biologically relevant.

**Table 2:** Summary of alanine-scanning mutagenesis for homologous contact residues in the α1 GlyR and α7 nAChR.

	*Strychnine*			*d-Tubocurarine*		
Ac-AChBP	α1 GlyR	IC_50_ (nM)	mut/wt	α7 nAChR	IC_50_ (µM)	mut/wt
	wt	46.8±8.75		Wt	1.19±0.17	
*Principal side*						
*loop A*						
Y91	A101F	583±47.6	12	Y93A	7.81±0.98	6.6
*loop B*						
K141				K145A	*NC*	
S144	S158A	13,800±1,410	294	S148A	176±10.8	148
W145	F159A	*NC*		W149A	*NC*	
*loop C*						
Y186	F207A^a^	5.59±1.89 mM	119,444	Y188A	*NC*	
C188	C209A	*NC*		C190A	*NC*	
C189	I210A	103±19	2.2	C191A	*NC*	
E191				E193A	8.10±0.68	6.8
Y193	F214A	*NC*		Y195A	*NC*	
*Complementary side*						
*loop D*						
T34	F44A^a^	130±4.06	2.8	S36A	2.25±0.52	2
Y53	F63A	12,000±3,000^b^	256	W55A	16.7±0.94	14
Q55	R65A	160±50^b^	3.4	Q57A	*NC*	
R57	Q67A^a^	689±176	14.7			
*loop E*						
M114	L127A	376±69.2	8	Q117A	2.28±0.14	1.9
I116	S129A	358±144	8	L119A	9.19±2.16	7.7
*loop F*						
D162	Q177A	3.36±0.63	0.1	G167A	0.043±0.002	0.03

The two-electrode voltage clamp technique was used to measure the affinity of strychnine on *d-TC* for mutants of the α1 GlyR and α7 nAChR, respectively. Structure-based sequence alignments were calculated and residues homologous to those involved in ligand interactions in AChBP structures were mutated to Ala. The Ala residue at position 101 in the α1 GlyR (homologous to Y91 in Ac-AChBP) was mutated to F. NC, no current.

a Residues that form unique contacts with strychnine in double ligand occupancy mode.

b IC_50_-values obtained from Grudzinska et al. [23].

For *d-TC*, previous mutagenesis studies have been carried out either on the muscle-type nAChR [1] or 5-HT_3_R [5],[12]. The heteropentameric muscle-type nAChR contains two different binding sites for *d-TC* with a 100-fold difference in affinity, whereas differences between human and mouse 5-HT_3_R yield a more than 1,000-fold difference in affinity. Both model systems either complicate the mutational analysis or make it difficult to derive conclusions that can be generalized to other CLRs. Here, we chose the human α7 nAChR to characterize the effect of homologous contact mutations on the potency of *d-TC*. The α7 nAChR, which is also a target of *d-TC* [4], is closely related to Ac-AChBP and is an attractive model system to extrapolate the results obtained from our X-ray crystal structures. Fifteen potential contacts in α7 nAChR were mutated and seven of these yielded a non-functional receptor, most of which are mutants of loop C. Similar to strychnine, crucial contacts are localized in loop B (S148, homologous to S144-AChBP) and loop D (W55, homologous to Y53-AChBP), which display a 148-fold and 14-fold drop in *d-TC*-potency, respectively. Mutations in other loops of the α7 nAChR have moderate to no effect (S36A, Y93A, Q117A, L119A, and E193A—homologous to T34-, Y91-, M114-, I116-, and E191-AChBP). Two mutants, namely G167A in the α7 nAChR and Q177A in the α1 GlyR (homologous to D162-AChBP), showed a significant decrease in the IC_50_-values for *d-TC* or strychnine compared to the wild-type receptor (*p*<0.05). A possible explanation for this observation is that these ligand interactions are energetically unfavorable in the wild type receptors.

Together, these AChBP-directed mutagenesis analyses pinpoint crucial interactions of strychnine and *d-TC* to residues in loop B and loop D of the α1 GlyR and α7 nAChR. This indicates that strychnine and *d-TC* form similar interactions in different classes of CLRs, which parallels our observation from the X-ray crystal structures with AChBP.

### Molecular Dynamics Simulation of AChBP Complexes

X-ray crystal structures of AChBP provide static snapshots of a receptor that undergoes highly dynamic changes upon ligand binding [34]. To investigate the validity of AChBP crystal structures and their different ligand binding modes observed in this study, we simulated the dynamic behavior of AChBP complexes with strychnine and *d-TC* and compared the calculated ligand orientations with those observed in co-crystal structures of AChBP. Simulation of an interface occupied either by a single strychnine molecule or two strychnine molecules shows that the system quickly resolves to equilibrium, indicating that a thermodynamically equilibrated conformation is obtained. Introduction of the ligand into an equilibrated conformation of the unliganded AChBP demonstrates that an induced fit is obtained within 9 to 12 ns (available as Movie S1). Superposition of simulated conformations and X-ray crystal structures showed only subtle changes characterized by an RMSD = 1.6 Å for strychnine in single occupancy, 1.8 Å for strychnine in double occupancy, 1.4 Å for *d-TC* in binding mode 1, and 3.2 Å for *d-TC* in binding mode 2 (Figure S2, panel A, B, and C).

Additionally, the system equilibrium was evaluated for each simulation as described in [35]. The equilibrium timeframe was truncated by 3 ns from the beginning along the time coordinate to avoid boundary artifacts. Interaction energy was measured for the ligand using thermodynamic integration in one direction along the time coordinate, giving 12±1 kJ/mol for the single strychnine conformation, 2×10±1 kJ/mol for double strychnine conformation, 11±1 kJ/mol for *d-TC* binding mode 1, and 10±1 kJ/mol for binding mode 2.

We attributed significant differences of the *d-TC* ligand pose 2 from others in RMSD from crystal structure to failure of H-bond formation with residue K141 from the (+) interface, which is observed in the X-ray structure. To investigate this we performed a more precise QM/MM (B3LYP/CHARMM) simulation. Details on system setup and simulation procedure are given in the Text S1 (see also Figure S3). After a 40 ps simulation RMSD from the crystal structure for the *d-TC* ligand pose 2 dropped to around 1.8 Å (Figure S2D).

**Figure 5:**
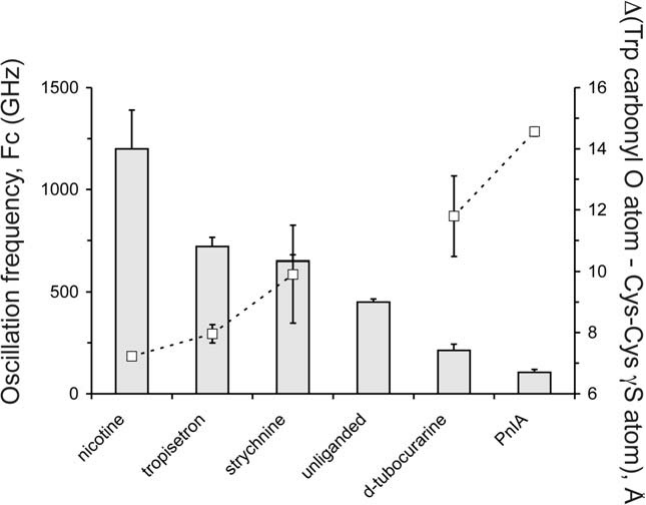
Correlation between a fast oscillatory movement of AChBP and C-loop closure. Analysis of the oscillatory frequency (*F*_c_) that describes the movement of AChBP during a molecular dynamic simulation for complexes with strychnine, *d-TC*, and typical agonists and antagonists for the nAChR. Complexes with agonists (nicotine) show a higher oscillation frequency (shown as grey bars) than antagonists (*d-TC* and PnIA). A good correlation exists with C-loop closure (same data as in Figure 3A, shown as white squares). No data are shown for C-loop closure of the unliganded state because the C-loop was disordered in our X-ray crystal structure of the apo form (pdb code 2w8e).

Finally, we measured the fluctuation properties of the equilibrium state using a direct Fourier transform. Fourier spectra and the resulting frequency characteristic (*F*_c_) were derived for AChBP bound to *d-TC* and strychnine and compared to typical agonists and antagonists. The primary frequency characteristic for unliganded AChBP was located at 450±15 GHz, whereas the complex with *d-TC* exhibited a leftward shift to 212±30 GHz, indicative of lower oscillation energy (Figure 5). This shift can be interpreted as a decrease in the thermodynamic temperature, which leads to an increased stability of the complex. A comparable leftward shift (*F*_c_ = 105±15 GHz) was observed in a control simulation performed for AChBP in complex with a variant of α-conotoxin PnIA (pdb code 2br8). In contrast, AChBP in complex with nicotine (pdb code 1uw6) demonstrated an extreme rightward shift (*F*_c_ = 1,200±550 GHz). The partial agonist tropisetron also demonstrated a slight increase in oscillation frequency (*F_c_* = 745±50 GHz). These data suggest a correlation between a leftward shift of *F*_c_ and antagonistic action of the ligand and a rightward shift and agonist activity. This increase in thermodynamic temperature upon agonist binding agrees well with previous findings that agonist potency is correlated with increase in domain mobility upon ligand binding [31],[36] and indicates a higher flexibility of AChBP compared to complexes with antagonists. Unexpectedly, the AChBP-complex with strychnine exhibits a small increase in oscillation frequency up to 655±30 GHz (Figure 5). This result parallels our analysis of the conformational states of loop C (Figure 3), showing that strychnine stabilizes loop C in a conformation similar to some agonists.

In summary, we found that deviations of the crystal structures from the simulated energy minimum are relatively small. All ligand conformations induce receptor fit with high energy, thereby increasing binding efficacy and reducing the probability to switch from one conformation to another. Analysis of the oscillatory movement of AChBP shows that agonist binding results in a thermodynamic destabilization of the receptor, whereas antagonist binding freezes AChBP in a state with a lower oscillation frequency. In conclusion, using molecular dynamics we observe distinct binding modes that are present in the AChBP model system and that might correspond to binding poses present in CLRs.

## Discussion

Acetylcholine binding protein (AChBP) has proven a valuable tool for structural studies with more than 20 prototypical ligands for the nAChR. In this study, we take advantage of the molecular recognition by AChBP of strychnine, a prototypical antagonist for the GlyR, and *d*-*TC*, which acts on the nAChR and 5-HT_3_R. We determined X-ray crystal structures of strychnine or *d-TC* complexes with AChBP and quantified the energetic contribution of observed interactions using molecular dynamic simulation and mutagenesis in α1 GlyR and α7 nAChR. Our results demonstrate that AChBP binds strychnine and *d-TC* with high affinity and serves as an appropriate model for mapping the binding site topography in different CLRs, including the GlyR, nAChR, and 5-HT_3_R.

In the AChBP complex with strychnine we observed that four binding pockets are fully occupied by a single ligand in identical binding orientations. A fifth binding pocket, which is also involved in a crystal contact, is occupied by two strychnine molecules in a different binding orientation. The relevance of the double occupancy in the binding sites of AChBP to true CLRs is not entirely clear. An intriguing possibility is that double ligand occupancy, which occurs at an interface between two neighboring AChBP molecules, may also occur at synapses where native CLRs are tightly clustered. In analogy, four molecules of epibatidine were suggested to be present in the muscle-type nAChR with its two expected binding sites [37].

In the AChBP complex with *d-TC* we observed that a preferred ligand orientation occurs in most binding pockets, but with varying degrees of occupancy. Two other binding pockets contain a ligand in a different orientation at full occupancy or a ligand that appears to switch between 2 orientations in a single site. Consequently, these different ligand binding modes result in varying conformations of loop C and stabilize the homopentameric AChBP in a structurally asymmetric state. This adds a new level of diversity among CLRs, whose heterogeneity is known to arise from homomeric and heteromeric assemblies of α- and non-α subunits with different pharmacological properties. Multiple orientations of the same ligand in binding sites of the same AChBP molecule revealed in our work may be present in complexes with true CLRs [38].

Our energy calculations of AChBP complexes, in combination with mutagenesis experiments on the α1 GlyR and α7 nAChR, point to crucial interactions with residues in loop A (Y91-AChBP), B (S144- and W145-AChBP), and D (Y53-AChBP). Gao et al. [24] investigated *d-TC* and metocurine binding modes using computational methods available at that time and proposed residues Y89 (loop A), W143 (loop B), Y192 (loop C), and L112 and M114 (loop E) in *Lymnaea* AChBP to be structural determinants of *d-TC* binding. Grudzinska et al. [23] simulated docking of strychnine into a homology model of the α1 GlyR and identified several residues crucial for strychnine-affinity, including F63 (loop D) and R131 (loop E). Some of these ligand contacts identified in both studies are confirmed by our results, but the overall ligand orientation of the strychnine and *d-TC* molecules modeled using computational approaches differs from those observed in our X-ray crystal structures and MD simulations. Moreover, we have systematically mutated the homologous contact residues in all loops of the α1 GlyR and α7 nAChR, based on ligand binding poses experimentally observed in X-ray crystal structures of AChBP. This allowed us to derive a common mode of action that defines poor selective recognition of strychnine and *d-TC* by various CLRs.

The essential residues in loop A, B, and D as identified in our study belong to the aromatic residues that are highly conserved among the CLR family, possibly explaining the wide range of CLRs that can be targeted by strychnine and *d-TC*. In contrast, peptide neurotoxins form an overlapping but more extended range of interactions with the principal and complementary faces of the binding site. This is clear upon comparison with the subtype-specific antagonist of nAChRs, α-conotoxin ImI. However, peptide toxins and smaller antagonists like *d-TC* and strychnine share a similar molecular mechanism of action: both classes of antagonists stabilize loop C in a similar extended conformation. Notably, for the ligands characterized in this study there is a significant correlation between C-loop closure observed in AChBP crystal structures, molecular dynamics equilibrium, and the frequency that characterizes AChBP oscillation. Thus, we propose that the conformation of loop C and the induced oscillation of the extracellular domain arise from residue interactions in the ligand-binding site. Combined, the effects on C-loop extension reflect the intrinsic properties of any given ligand and therefore predict well its type of action. We suggest that antagonist effects are transmitted through a thermodynamic stabilization of the extracellular domain and arise from a limited range of residue contacts as shown for strychnine and *d-TC*. These defined ligand-receptor interactions are found for homologous residues in different CLRs and likely explain the low selective antagonism of strychnine and *d-TC*.

## Materials and Methods

### Co-Crystallization of AChBP with Strychnine and d-TC

*Aplysia* AChBP was expressed and purified from Sf9 insect cells as previously published [25]. Strychnine and *d-TC* were obtained from Sigma and co-crystallized at a concentration of 1–5 mM. Crystals for Ac-AChBP+ strychnine were grown in 200 mM sodium acetate, 100 mM bistrispropane at pH 8.5, 15.5% PEG3350. Crystallization conditions for Ac-AChBP+*d-TC* were 200 mM Na_2_SO_4_, 100 mM bistrispropane at pH 8.5, 15% PEG3350. Growth of crystals at 4 °C was essential to obtain good diffraction for both complexes. Cryoprotection was achieved by adding glycerol to the mother liquor in 5% increments to a final concentration of 30%. Crystals were flash-cooled by immersion in liquid nitrogen. Diffraction data processing was done with MOSFLM and the CCP4 program suite. The structure was solved by molecular replacement using MOLREP. Automated model building was carried out with ARP/wARP and refinement was done with REFMAC or PHENIX with TLS and NCS restraints. Manual building was done with COOT and validation of the final model was carried out with MOLPROBITY. All model figures were prepared with PYMOL.

### Pharmacological Characterization of AChBP, Mutant α1 GlyR and α7 nAChRs

Competitive binding assays with ^3^H-epibatidine were carried out as previously published with minor modifications (see Text S1). Electrophysiological assays were carried out using the two-electrode voltage clamp technique. The cDNA encoding human α1 GlyR was subcloned into pGEM-HE for oocyte expression with a PCR strategy and verified by sequencing. The cDNA was linearized with *Nhe*I and transcribed with the T7 mMessage mMachine kit from Ambion. The cDNA encoding the human α7 nAChR was cloned into pMXT and linearized with *Bam*HI for transcription with the SP6 mMessage mMachine kit from Ambion. All mutants were engineered using a Quikchange method (Stratagene) and verified by sequencing. Recordings were obtained from oocytes 2–5 d after injection with 50 nl of ∼1 ng/nl RNA. For the determination of IC_50_-values varying concentrations of strychnine or *d-TC* were co-applied with EC_50_-concentrations of glycine or acetylcholine, respectively. Peak current responses in the presence of increasing concentrations of strychnine or *d-TC* were averaged and the mean ± s.e.m. analyzed by non-linear regression using a logistic equation (GraphPad Prism 5). Student's *t* test was used for statistical comparison of paired observations.

### Molecular Dynamics and Docking Simulations

Sequence analysis was performed with the ClustalW2 algorithm. Homology models of the α1 GlyR and α7 nAChR were used to verify our mutagenesis strategy, which was based on predictions from sequence alignments. Modeller9v6 was used without any GUI software and protein structures with pdb code 2vl0 and 2bg9 as templates. Energy for all models was minimized with CHARMM27. Docking was performed in AUTODOCK4 with 32 flexible bonds in receptor selected from residues inside a sphere r = 5Å centered at mass center of the ligand in the corresponding X-ray structure. A full description of molecular dynamic simulation methods is given in Text S1. Molecular analysis and visualization was performed in UCSF Chimera and PyMol. For video editing AVS Video Editor was used.

### Accession Numbers

Protein Data Bank accession codes for previously published X-ray crystal structures are: Ac-AChBP in complex with lobeline (2bys), epibatidine (2byq), imidacloprid (3c79), thiacloprid (3c84), HEPES (2br7), polyethyleneglycol (2byn), DMXBA (2wnj), 4OH-DMXBA (2wn9), cocaine (2pgz), anabaseine (2wnl), *in silico* compound 31 (2w8f), MLA (2byr), *in silico* compound 35 (2w8g), sulfates (3gua), α-conotoxin ImI (2c9t and 2byp), α-conotoxin PnIA variant (2br8), α-conotoxin TxIA (2uz6), apo state (2w8e), Y53C-MMTS (2xz5) with acetylcholine, Y53C-MTSET (2xz6). Bt-AChBP in complex with CAPS (2bj0) and Ls-AChBP in complex with nicotine (1uw6), clothianidin (2zjv), HEPES (1ux2), carbamylcholine (1uv6), imidacloprid (2zju), α-cobratoxin (1yi5). Monomeric extracellular domain from mouse α1 nAChR in complex with α-bungarotoxin (2qc1).

Structures of Ac-AChBP in complex with strychnine (2xys) and *d*-tubocurarine (2xyt) were obtained during this study.

PubChem coordinates: strychnine (441,071) and *d*-tubocurarine (6,000).

## Supporting Information

Figure S1 Topology of the AChBP binding pocket and homologous residues in other CLRs. (A) Surface representation of the AChBP binding pocket. Residues from loop C (185–193) were omitted for clarity. Ligand contacts that are common to strychnine and *d*-tubocurarine were color coded according to importance in the mutagenesis analysis in Table 2: essential (red), important (green). and less important (blue). (B) Sequence alignment of AChBPs, glycine receptors, and nicotinic acetylcholine receptors. Structure-based sequence alignments were calculated using secondary structure matching (SSM) for AChBPs from different species and the structure for the monomeric mouse α1 nAChR extracellular domain [39]. This alignment was separately seeded with human GlyR/GABA_A_R and nAChRs/5-HT_3_R sequences, respectively, and aligned in ClustalW. Both alignments were then merged with manual adjustments for loop F and loop C. Amino acids involved in ligand-receptor contacts in the strychnine- and *d*-tubocurarine-bound structures are indicated in black. In our study, homologous positions in human α1 GlyR and human α7 nAChR were mutated to alanine, except for α1 GlyR Ala101, which was mutated to phenylalanine. (1) indicates residues that only form contacts with strychnine, and (2) indicates a residue that only forms contacts with *d*-tubocurarine.
(TIF)

Figure S2 Panels A and B compare the AChBP conformations after energetic minimization with molecular dynamic simulation for strychnine complexes with single occupancy (A) and double occupancy (B). The unliganded protein equilibrium state is shown in cyan, X-ray crystal structure in magenta, liganded equilibrium state in yellow. Panels C and D compare the AChBP simulated conformations for d-tubocurarine complexes with binding mode 1 (C) and mode 2 (D). The same color codes are used as in (A) and (B).
(TIF)

Figure S3 Setup of classical simulations. Protein was simulated as a complete pentamer with a single ligand bound. A 9 nm^3^ solvation cube consisting of 38,256 SPC water molecules was applied centered at the center of the protein (halfway from upper lumen at the axis of radial symmetry).
(TIF)

Movie S1 Induced fit of strychnine and d-tubocurarine in *Aplysia* AChBP.
(MOV)

Table S1 Crystallographic and model refinement statistics.
(PDF)

Table S2 Comparison of residue contacts observed in crystal structures of AChBP in complex with α-conotoxin ImI, strychnine, *d*-tubocurarine, and nicotine. (*) indicates residues that are unique contacts for strychnine in double occupancy of the binding pocket. (#) contact residues for the nicotine bound-structure of *Lymnaea* AChBP are indicated according to homologous residues for *Aplysia* AChBP (first column).
(PDF)

Text S1 Supplementary methods.
(DOC)

## Abbreviations

5-HT_3_R: serotonine 3 receptor
AChBP: acetylcholine binding protein
CLR: cys-loop receptor
*d-TC*: *d*-tubocurarine
GABA: γ-aminobutyric acid
GlyR: glycine receptor
nAChR: nicotinic acetylcholine receptor
