# Supplementary figures and images for: A Structural and Mutagenic Blueprint for Molecular Recognition of Strychnine and d-Tubocurarine by Different Cys-Loop Receptors
Source: PLoS Biol. 2011 Mar 29;9(3):e1001034. doi: 10.1371/journal.pbio.1001034 (PMC3066128; doi:10.1371/journal.pbio.1001034)

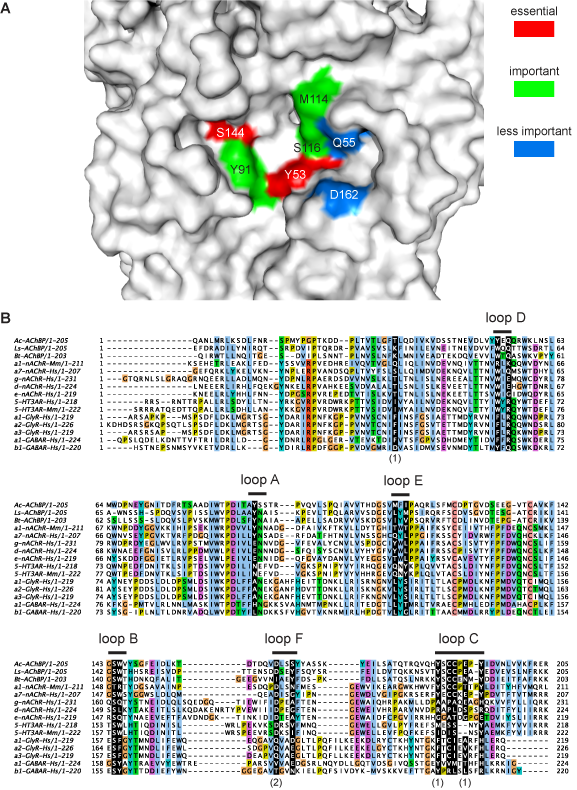

Supplement: Figure S1 — Topology of the AChBP binding pocket and homologous residues in other CLRs. (A) Surface representation of the AChBP binding pocket. Residues from loop C (185–193) were omitted for clarity. Ligand contacts that are common to strychnine and d-tubocurarine were color coded according to importance in the mutagenesis analysis in Table 2: essential (red), important (green). and less important (blue). (B) Sequence alignment of AChBPs, glycine receptors, and nicotinic acetylcholine receptors. Structure-based sequence alignments were calculated using secondary structure matching (SSM) for AChBPs from different species and the structure for the monomeric mouse α1 nAChR extracellular domain [39]. This alignment was separately seeded with human GlyR/GABAAR and nAChRs/5-HT3R sequences, respectively, and aligned in ClustalW. Both alignments were then merged with manual adjustments for loop F and loop C. Amino acids involved in ligand-receptor contacts in the strychnine- and d-tubocurarine-bound structures are indicated in black. In our study, homologous positions in human α1 GlyR and human α7 nAChR were mutated to alanine, except for α1 GlyR Ala101, which was mutated to phenylalanine. (1) indicates residues that only form contacts with strychnine, and (2) indicates a residue that only forms contacts with d-tubocurarine. (TIF) [file pbio.1001034.s001.tif]

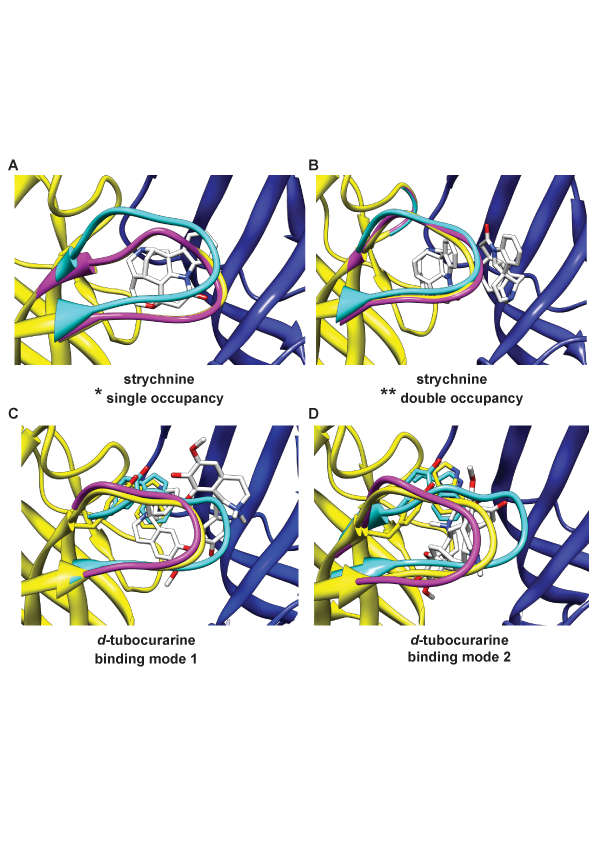

Supplement: Figure S2 — Panels A and B compare the AChBP conformations after energetic minimization with molecular dynamic simulation for strychnine complexes with single occupancy (A) and double occupancy (B). The unliganded protein equilibrium state is shown in cyan, X-ray crystal structure in magenta, liganded equilibrium state in yellow. Panels C and D compare the AChBP simulated conformations for d-tubocurarine complexes with binding mode 1 (C) and mode 2 (D). The same color codes are used as in (A) and (B). (TIF) [file pbio.1001034.s002.tif]

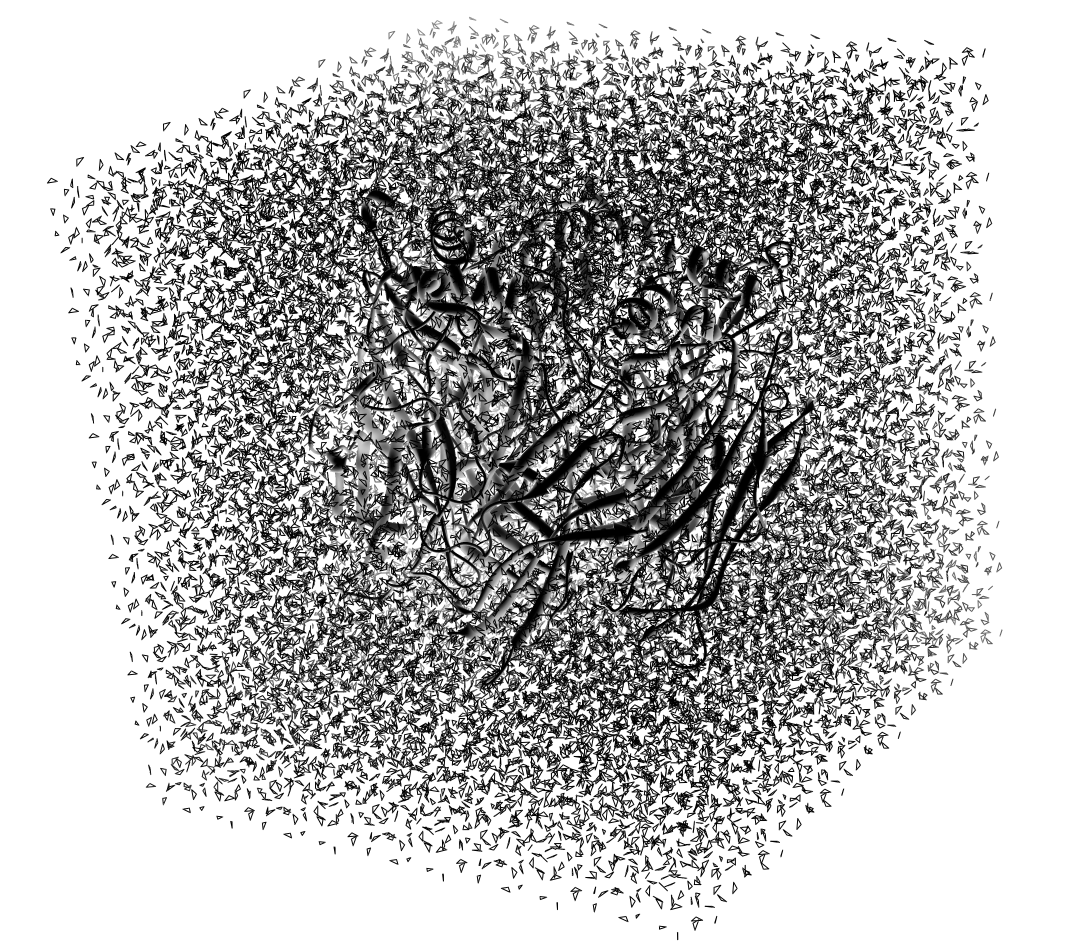

Supplement: Figure S3 — Setup of classical simulations. Protein was simulated as a complete pentamer with a single ligand bound. A 9 nm3 solvation cube consisting of 38,256 SPC water molecules was applied centered at the center of the protein (halfway from upper lumen at the axis of radial symmetry). (TIF) [file pbio.1001034.s003.tif]
