# Supplementary material for: A Structural and Mutagenic Blueprint for Molecular Recognition of Strychnine and d-Tubocurarine by Different Cys-Loop Receptors
Source: PLoS Biol. 2011 Mar 29;9(3):e1001034. doi: 10.1371/journal.pbio.1001034 (PMC3066128; doi:10.1371/journal.pbio.1001034)
Supplement: Table S1 — Crystallographic and model refinement statistics. (PDF) [file pbio.1001034.s005.pdf]

	Ac-AChBP + strychnine	Ac-AChBP + *d*-tubocurarine
<u>Crystallographic statistics</u>		
Spacegroup	$P4_12_12$	$P2_12_12_1$
a,b,c (Å)	100.59, 100.59, 289.68	83.34, 137.65, 236.42
α, β, γ (°)	90, 90, 90	90, 90, 90
Resolution limits (Å)	49.56 - 1.91 (2.01 - 1.91)	54.31 - 2.00 (2.11 - 2.00)
Rmerge (%)	8.4 (61.6)	8.4 (64.7)
I/σ	7.6 (1.3)	5.8 (1.0)
Multiplicity	5.6 (5.4)	7.4 (7.4)
Completeness (%)	98.2 (91.7)	99.8 (99.9)
Total number of reflections	634459 (20815)	1355056 (196693)
Number unique reflections	113796 (3852)	183396 (26557)
Wilson B factor	23.01	29.22
<u>Refinement and model statistics</u>		
R_{work} (%)	17.18	17.59
R_{free} (%)	20.97	22.01
Rmsd bond distance (Å)	0.007	0.007
Rmsd bond angle (°)	1.194	1.092
Average B-factors		
protein	26.24	35.12
ligand binding mode 1	30.23	30.67
ligand binding mode 2	60.67	63.82
ligand binding mode 3		47.41
water	37.44	40.34
