# Supplementary material for: A Structural and Mutagenic Blueprint for Molecular Recognition of Strychnine and d-Tubocurarine by Different Cys-Loop Receptors
Source: PLoS Biol. 2011 Mar 29;9(3):e1001034. doi: 10.1371/journal.pbio.1001034 (PMC3066128; doi:10.1371/journal.pbio.1001034)
Supplement: Table S2 — Comparison of residue contacts observed in crystal structures of AChBP in complex with α-conotoxin ImI, strychnine, d-tubocurarine, and nicotine. (*) indicates residues that are unique contacts for strychnine in double occupancy of the binding pocket. (#) contact residues for the nicotine bound-structure of Lymnaea AChBP are indicated according to homologous residues for Aplysia AChBP (first column). (PDF) [file pbio.1001034.s006.pdf]

	α-conotoxin ImI	strychnine	*d*-tubocurarine	nicotine [#]
Principal side				
loop A				
Y91	✓	✓	✓	✓
K141			✓	
loop B				
S144	✓	✓	✓	
W145	✓	✓	✓	✓
V146	✓			✓
Y147	✓			
loop C				
Y186	✓	✓ [*]	✓	
C188	✓	✓	✓	✓
C189	✓	✓	✓	✓
E191	✓		✓	
Y193	✓	✓	✓	
I194	✓			
Complementary side				
loop D				
T34		✓ [*]	✓	
Y53	✓	✓	✓	✓
Q55	✓	✓	✓	
R57	✓	✓ [*]		
loop E				
D75	✓			
R77	✓			
V106	✓			
T108	✓			
M114	✓	✓	✓	✓
I116	✓	✓	✓	✓
loop F				
D162	✓	✓		
S164	✓			
S165		✓	✓	
