# Supplementary material for: A Structural and Mutagenic Blueprint for Molecular Recognition of Strychnine and d-Tubocurarine by Different Cys-Loop Receptors
Source: PLoS Biol. 2011 Mar 29;9(3):e1001034. doi: 10.1371/journal.pbio.1001034 (PMC3066128; doi:10.1371/journal.pbio.1001034)
Supplement: Text S1 — Supplementary methods. (DOC) [file pbio.1001034.s007.doc]

**Supplementary methods**

*Radioligand binding assays*

Human neuroblastoma cells (SH-SY5Y) stably expressing human α7 nAChR were obtained from Christian Fuhrer (Department of Neurochemistry, Brain Research Institute, University of Zurich). The cells were maintained in DMEM/F12 (Dulbecco's Modified Eagle Medium / Nutrient Mixture F-12) supplemented with 10% fetal calf serum (FCS), 50 IU/mL penicillin, 50 µg/mL streptomycin and 0.1 mg/ml geneticin in 5% CO2 humidified atmosphere at 37°C. Batches of cells from several passages were washed 3x with PBS, harvested and after centrifuging aliquots, the cell-pellets were stored at -80°C until use. Although there appeared to be little batch-to-batch variation, only one passage was used for the triplicate experiments as described below. Competition binding assays were performed in buffer (PBS, 20 mM Tris, 0.05% Tween, pH 7.4) in a final assay volume of 100 µl. ^3^H-epibatidine (GE Healthcare, specific activity 56 Ci/mmol) was used as radioligand at 2.23 nM. Ligand was added and 2-30 ng His-tagged Ls- or Ac-AChBP, followed by the addition of 200 µg PVT Copper His-Tag SPA beads (Perkin-Elmer) and incubation for 1.5 h at room temperature under continuous shaking. The SPA beads were allowed to settle for 2-4 h and the radioactivity was measured in a Wallac 1450 MicroBeta liquid scintillation counter. All radioligand binding data were evaluated by a nonlinear, least-squares curve fitting procedure using Graphpad Prism (version 5.0a, GraphPad Software, Inc., San Diego, CA). All data are represented as the mean ± SEM from at least three independent experiments.

Competition binding assays on human α7 nAChRs were performed in buffer (PBS, 20 mM Tris, 0.05% Tween, pH 7.4) in a final assay volume of 100 µl. ^3^H-MLA (American Radiolabeled Chemicals, Inc, specific activity ~ 100 Ci/mmol) was used at 1.8 nM (which is at the K_D_) and added to the ligands. Frozen cell pellets with the human α7 nAChR were thawed, homogenized in ice-cold binding buffer, sonicated before use and added at 0.1 mg/ml. After incubation for 1.5 h at room temperature under continuous shaking, bound radioligand was collected on 0.3 % polyethyleneimine-pretreated Unifilter-96 GF/C filter-plates (Perkin Elmer) and washed with in ice-cold 50 mM Tris buffer, pH 7.5. After drying the filter-plates, scintillation fluid (MicroScint, Perkin Elmer) was added and the radioactivity was measured as above.

*Molecular dynamics and docking simulations*

All molecular dynamics were performed in GROMACS4 package CHARMM27 force field (45) for 50 ns under canonical (NVT) ensemble using LJ-4 potential form and Nose-Hoover thermostat. Each system was simulated in two condition sets reflecting crystallization and physiological conditions in respect to ionic force and specific ion concentrations. As divergence between two conditions sets was below the X-ray structure resolution, only physiological conditions set was used for obtaining data. Initially we built a system with a dimer, a single binding site with a ligand molecule bound and a set of water molecules. After 10 ps simulation all water molecules further then 2.2Å from a protein atom were removed. To investigate probable long-range interactions between binding sites, we shifted to simulating complete pentamers; only one ligand bound (the pose investigated in this simulation) was left. The system was simulated as 9 nm^3^ solvation cube consisting of 38256 SPC water molecules. Periodic boundary conditions were not used. A figure depicting solvated systems is included in supplementary material (supplementary figure 3). Electrostatic interactions were treated with particle-mesh Ewald (PME) method with distance cutoff 10Å. For induced fit simulation first the apo-protein was simulated under physiological conditions for 10 ns. Then simulation was stopped and a pre-protonated ligand was introduced artificially into the presumed binding site. System was then let to resolve for 40 ns, adopting a conformation very close, but not completely similar to one observed in crystal structures. The overlay of these conformations is given in Figure 4. To get an average and evaluate the equilibrium state we used method proposed in Schiferl and Wallace (J. Chem. Phys., 1985) in variation employed by Senn et al. (J. Chem. Theory Comput., 2005). Energy measurement was performed using thermodynamic integration along the simulation time coordinate as in Senn et al., but in one direction. To analyze fluctuation rates and derive a primary frequency characteristic of the system we used Fast Fourier transform (Frigo and Johnson, Proceedings of the IEEE, 2005) applied to RMSD(t) of the equilibrium phase proven as described for the whole protein. In-depth description of the approach together with its application to other MD systems will be published elsewhere.

QM/MM simulations were performed using ChemShell platform (Sherwood et al., J. Mol. Struct., 2003) as coupler and MD engine with Turbomole code (Ahlrichs et al., Chem. Phys. Lett., 1989). QM part was treated at B3LYP/6-31G level and MM part with CHARMM27. In each simulation only ligand was treated as QM region. A box with a=9Å centered on the C1 of the *d-TC* ring 1 was included into the MM region. We used QM/MM setup described in Senn et al. as a template and simulation time 40 ps. We employed no electrostatic cutoff for QM/MM system. An electronic embedding scheme scheme (Bakowies and Thiel, J. Phys. Chem., 1996) incorporating the MM charges into the one-electron Hamiltonian of the QM treatment and hydrogen link atoms with charge shift model for the QM/MM boundary were adopted in the QM/MM treatment. MD simulations were performed as constant-temperature 300K under NVT ensemble and Nose-Hoover thermostat to control system temperature.

References:

Schiferl, S. K., Wallace, D. C., *J. Chem. Phys*. 1985, 83, 5203.

Frigo, M., Johnson, S.G., The Design and Implementation of FFTW3, *Proceedings of the IEEE* 2005, 93: 216–231.

Bakowies, D., Thiel, W. Hybrid Models for Combined Quantum Mechanical and Molecular Mechanical Approaches *J. Phys. Chem.*, 1996, 100 (25), 10580–10594.

Ahlrichs, R., Bar, M., Haser, M., Horn, H., Kolmel, C. *Chem. Phys.*

*Lett.* 1989, 162, 165

Sherwood, P. et al. *Theochems - J. Mol. Struct*. 2003, 632, 1.

Brooks, B.R.,Bruccoleri,R.E., Olafson,B.D., States, D.J., Swaminathan, S., Karplus, M.J. *Comput. Chem.* 1983, 4, 187.
